# Do changes in the frailty score differ by the type of group sports and exercises participated in? A 3-year longitudinal study

**DOI:** 10.1186/s11556-024-00342-x

**Published:** 2024-03-20

**Authors:** Taishi Tsuji, Satoru Kanamori, Ryota Watanabe, Meiko Yokoyama, Yasuhiro Miyaguni, Masashige Saito, Katsunori Kondo

**Affiliations:** 1 Institute of Health and Sport Sciences, University of Tsukuba, 3-29-1 Otsuka, Bunkyo City, Tokyo 112-0012 Japan; 2 Center for Preventive Medical Sciences, Chiba University, 1-33 Yayoi-Cho, Inage Ward, Chiba City, Chiba 263-8522 Japan; 3 Teikyo University Graduate School of Public Health, 2-11-1 Kaga, Itabashi City, Tokyo 173-8605 Japan; 4 Department of Preventive Medicine and Public Health, Tokyo Medical University, 6-1-1 Shinjuku, Shinjuku City, Tokyo 160-8402 Japan; 5 Center for Well-Being and Society, Nihon Fukushi University, 5-22-35 Chiyoda, Naka Ward, Nagoya City, Aichi 460-0012 Japan; 6 Center for Gerontology and Social Science, Research Institute, National Center for Geriatrics and Gerontology, 7-430 Morioka-Cho, Obu City, Aichi 474-8511 Japan; 7 Department of Social Welfare, Nihon Fukushi University, Okuda, Mihama-Cho, Aichi, Chita-Gun 470-3295 Japan

**Keywords:** Kihon Checklist, Comprehensive geriatric assessment, Social participation, Propensity score, Walking

## Abstract

**Background:**

Older adults who engage in group sports and exercises achieve greater health benefits than those who exercise by themselves. The benefits of group participation may vary depending on the type of sports/exercise they engage in. The present study aimed to identify the association between specific sports and exercise types performed in groups and evaluate the longitudinal changes in multidimensional frailty scores among community-dwelling older adults in Japan.

**Methods:**

We used 3-year follow-up data from the Japan Gerontological Evaluation Study and analyzed 33,746 men and 36,799 women aged ≥ 65 years. To elucidate the relationship between participation in 20 types of group sports/exercises in 2016 (baseline) and the change in frailty score (using the Kihon Checklist, KCL) from 2016 to 2019, we performed linear regression analyses through multivariate adjustments for age group, self-rated health, marital status, living alone, occupational status, years of education, alcohol drinking status, smoking status, equivalent income, and disease status using an inverse probability weighting method. *P* < 0.05 was considered statistically significant.

**Results:**

The mean change in KCL scores over 3 years was + 0.62 and + 0.61 points in men and women, respectively, implying the degree of frailty worsened. The sports/exercise types that significantly prevented increments in KCL scores for both sexes compared to non-participants were hiking (men: *B*, − 0.36; women: *B*, − 0.29), walking (men: *B*, − 0.26; women: *B*, − 0.24), tennis (men: *B*, − 0.23; women: *B*, − 0.24), ground golf (men: *B*, − 0.21; women: *B*, − 0.19), and weight exercises (men: *B*, − 0.19; women: *B*, − 0.16).

**Conclusion:**

Participation in specific sports and exercise groups offer significant physical and psychological benefits for frailty prevention among older adults in Japan. The results of this study may offer substantive evidence to encourage older adults to participate in group activities for the prevention of multidimensional frailty. It will also help public health stakeholders to decide which type of sports and exercise groups to promote in a community.

**Supplementary Information:**

The online version contains supplementary material available at 10.1186/s11556-024-00342-x.

## Background

Participation in group sports and exercises is known to reduce the risk of functional decline [1], depressive symptoms [2], and falls [3] among older adults compared to participation in individual ones. The additive effects of such group activities influence health outcomes through various mechanisms which involve the benefits of physical activity (such as ensuring good compliance and longer durations of activity), the psychological effects of group participation (ensuring enjoyment, enhancing self-esteem, and decreasing stress), and the social implications of group sports (such as receiving social support, social capital, and social influence) [4]. Interestingly, a previous study confirmed that the socio-psychological health benefits obtained from different group sports and exercises depend on the specific sport and exercise in which older adults participate. A three-year follow-up of community-dwelling older adults’ participation in 20 different sports and exercise groups and its association with the changes in depressive symptoms, self-rated health, subjective well-being, and frequency of laughter found that participation in a golf group for older men was positively associated with the aforementioned outcomes, and engaging in walking, weight exercises, or hiking groups by older women effectively relieved their depressive symptoms and improved their self-rated health [5]. However, the relationship of participation in different types of group sports and exercises with comprehensive functioning, including factors other than socio-psychological health (such as physical function, cognitive function, and nutritional status), which are crucial for older adults to undergo healthy aging, has not been explored.

Frailty is a clinical condition characterized by a decline in an individual’s ability to maintain homeostasis due to a combination of functional decline, disabilities, and diseases, leading to increased vulnerability to endogenous and exogenous stressors [6, 7]. Hitherto, it has been established that frailty evaluations that are based on a comprehensive geriatric assessment have better predictive ability for mortality than evaluations performed using only a specific functional decline, such as physical frailty [8]. On the contrary, it can be speculated that frailty prevention may benefit from participation in different sports and exercise activities, community organizations, clubs, and groups [9, 10].

Therefore, this study aimed to identify the associations between participation in 20 specific types of group sports and exercises and frailty score changes over 3 years based on a comprehensive geriatric assessment (comprising instrumental and social activities of daily living, physical function, nutritional status, oral function, cognitive function, and depressive mood) in community-dwelling older adults. We expected that there will be differences in their association with frailty based on the type of group sports and exercises and that the type closely associated with frailty will vary between men and women. Elucidating these can help determine which type of group sports and exercises is effective for preventing frailty in men and women.

## Methods

### Study design and participants

**Fig. 1:**
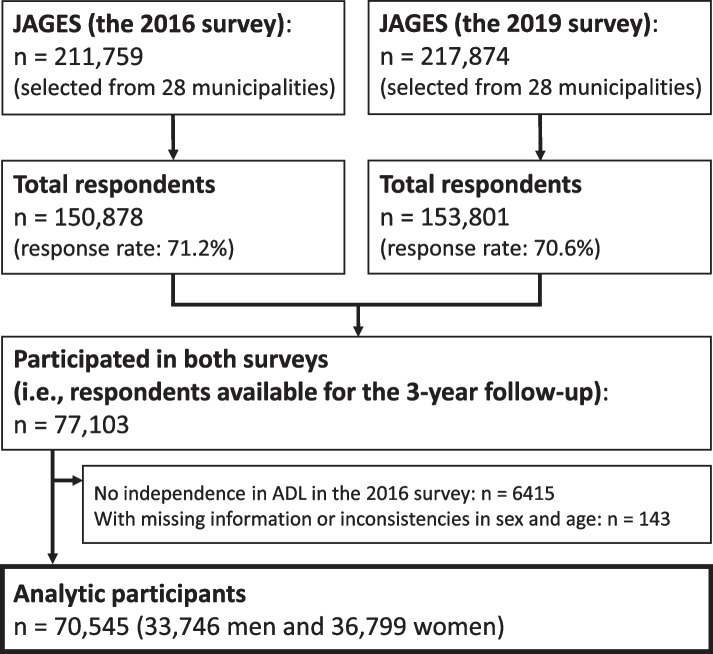
Flow diagram of the study participants. JAGES: Japan Gerontological Evaluation Study; ADL: activities of daily living

Figure 1 depicts a flow diagram of the study participants. We conducted a population-based 3-year longitudinal study in Japan using data from the Japan Gerontological Evaluation Study (JAGES). The JAGES is an ongoing cohort study exploring social, environmental, and behavioral factors related to the loss of health with regards to functional decline or cognitive impairment among older adults [11, 12]. Self-reported questionnaires were mailed to community-dwelling adults aged ≥ 65 years who were sampled from 28 municipalities, including metropolitan, urban, semi-urban, and rural communities from 14 prefectures in Japan as far as Hokkaido (the northernmost prefecture) and Kyushu (the southernmost region), between October and November 2016 (the 2016 survey) and between November 2019 and January 2020 (the 2019 survey). Both surveys were administered to all eligible residents in the 11 small municipalities and using random sampling in the 17 large municipalities. In the 2016 survey, the questionnaires were mailed to 211,759 people, with 150,878 of them responding (response rate: 71.2%). Likewise, in the 2019 survey, of the 217,874 people approached, 153,801 (70.6%) responded to the questionnaire. Out of the 77,103 respondents who participated in both surveys, 6415 people who had no independence in activities of daily living in the 2016 survey and 143 who had missing information or inconsistencies in sex and age data were excluded from the study.

For the present study, we used data from 70,545 eligible respondents (33,746 men and 36,799 women). The mean age was 73.1 ± 5.5.

### Multidimensional frailty score: Kihon Checklist (KCL)

The KCL is a simple self-reported survey developed by the Japanese Ministry of Health, Labour and Welfare to identify older adults at risk of requiring care/support. It consists of 25 questions regarding instrumental (3 questions) and social (4 questions) activities of daily living, physical functions (5 questions), nutritional status (2 questions), oral function (3 questions), cognitive function (3 questions), and depressive mood (5 questions) [13, 14]. With question being worth one point, the total score ranges from 0 to 25, and a higher score indicates greater frailty. The area under the receiver operating characteristics curves for the evaluation of frailty using the KCL was 0.81 for prefrailty and 0.92 for frailty [14]. Another study reported that with higher total scores, the risk of future functional decline and mortality increased proportionately [15].

### Participation in different types of group sports and exercises

At first, participants were asked about their frequency of sports club or group participation; those who participated at least once a year were further asked “what type of sports do you currently do in those clubs or groups (multiple answers possible)?” Possible responses for this question included 1) walking, 2) running and jogging, 3) fitness exercises, 4) weight exercises, 5) hiking, 6) golf, 7) ground golf, 8) gateball, 9) dance, 10) yoga, 11) aerobics, 12) petanque, 13) Tai Chi, 14) swimming, 15) aquatic exercises, 16) table tennis, 17) bowling, 18) bicycling, 19) tennis, and 20) other sports [5]. We also created a dichotomized variable for whether or not they participated in any of these types.

Fitness exercises are low-to-moderate-intensity exercises, such as calisthenics and seated exercises, which mainly focus on health promotion and social interaction. Ground golf, a new kind of golf that simplifies the golf game to promote lifelong sports, is widely popular among older adults in Japan [16]. Gateball, a team sport that originated in Japan, entails competing for points by hitting a ball against a target using a stick [17]. The details of the rules of these sports and how to play them are presented in the respective articles.

### Covariates

We evaluated different variables, which are reportedly associated with sports group participation in older adults, for their potential confounding effect on the association between group participation and frailty [18]. The effect of sex was controlled by conducting a stratified analysis. Data on the following variables were collected: age group (65–69, 70–74, 75–79, 80–84, and ≥ 85 years), marital status (married or unmarried), living alone (no or yes), occupational status (employed, retired, or never employed), number of years of education (≥ 13, 10–12, or < 10 years), alcohol consumption status (none, past, or current), and smoking status (none, past, or current). Self-rated health was measured by a 4-point Likert scale: very good, good, somewhat poor, or poor. Each participant’s annual equivalent income was calculated by dividing the household income by the square root of the number of household members and categorized into three groups: ≥ $40,000; $20,000–$39,999; or < $20,000 per year (1 dollar = 100 yen). Each participant’s disease status was assessed with “yes” or “no” responses and included hypertension, stroke, cardiovascular disease, diabetes mellitus, hyperlipidemia, musculoskeletal disorders, and cancer.

### Statistical analysis

We calculated descriptive statistics by sex. Linear regression analyses were performed to examine the association between the type of sports and exercise groups in the 2016 survey and the change in the frailty score from 2016 to 2019 (i.e., values obtained by subtracting the total KCL score in 2016 from that in 2019). The calculated change in score was approximately normally distributed and met one of the assumptions for the application of linear regression. The homogeneity of variances and linearity were likely to be acceptable in the large sample used in the present study. The unstandardized coefficient (*B*) with its 95% confidence interval (95% CI) was calculated individually for men and women. We then examined the interaction between each type and sex to investigate whether the association with changes in the frailty score differed according to sex. In these regression analyses, we conducted multivariate adjustment using an inverse probability weighting (IPW) method as the tool to adjust for differences between the two groups (i.e., participation or non-participation in each type of sport and exercise group) [19]. To adjust for the between-group differences in participant characteristics “Covariates” section), we developed propensity scores to estimate the probability that older adults would participate in each type of sport and exercise group by conducting a logistic regression analysis. This ensures a balanced approach and involves weighting each participant in each group by the inverse of the probability that they would participate in the group and weighting each participant who did not participate in the group by the inverse of the probability that they would not participate in the group. Additionally, we imputed incomplete variables using a multivariate normal imputation method. We created 20 imputed datasets using all variables introduced in the current analyses, after which the estimated parameters were combined using Rubin’s combination methods [20, 21]. We used Stata/MP version 17.0 (StataCorp, College Station, Texas, USA) for all statistical analyses, with the statistical significance set at *P* < 0.05.

## Results

Table 1 and Supplemental Table 1 summarize the descriptive data before multiple imputations were performed for missing values. Supplemental Fig. 1 depicts the mean KCL total score according to age in 2016. We found that, on average, men (+ 0.62 points) and women (+ 0.61 points) participants had higher KCL total scores (i.e., worsening frailty) in 2019 than in 2016. Among analyzed participants, 32.2% of men and 34.0% of women participated in some kind of sports and exercise group. The three most popular sport/exercise types were golf (11.8%), walking (8.3%), and ground golf (5.7%) among men, while fitness exercises (13.0%), walking (7.9%), and weight exercises (6.1%) were the most commonly performed sports/exercise types among women.

**Table 1:** Characteristics of participants by sex

	Men (*n* = 33,746)			Women (*n* = 36,799)		
	n	Mean	SD	n	Mean	SD
KCL total score (2016)	30,302	3.96	3.08	31,501	3.72	3.02
Δ KCL total score (2016–2019)						
All participants	25,806	0.62	2.77	25,938	0.61	2.57
Not participating in any of group	14,601	0.63	2.86	13,002	0.61	2.70
Participating in any of group	8779	0.57	2.51	9524	0.59	2.34
Missing	2426			3412		
	n	%		n	%	
Types of sports and exercise groups (multiple answer)						
Fitness exercises	959	2.8		4785	13.0	
Walking	2803	8.3		2919	7.9	
Golf	3964	11.8		457	1.2	
Weight exercises	1297	3.8		2,243	6.1	
Ground golf	1911	5.7		1467	4.0	
Swimming	617	1.8		1143	3.1	
Dance	306	0.9		1389	3.8	
Hiking	922	2.7		748	2.0	
Yoga	160	0.5		1424	3.9	
Table tennis	560	1.7		965	2.6	
Aquatic exercises	350	1.0		905	2.5	
Tai Chi	244	0.7		943	2.6	
Tennis	642	1.9		362	1.0	
Aerobics	121	0.4		657	1.8	
Running/Jogging	554	1.6		197	0.5	
Bowling	386	1.1		289	0.8	
Gateball	207	0.6		169	0.5	
Bicycling	149	0.4		42	0.1	
Petanque	102	0.3		86	0.2	
Other	1602	4.8		1173	3.2	
(Any of)	10,853	32.2		12,494	34.0	
Missing	3757	11.1		6021	16.4	
Age groups (years)						
65–69	11,157	33.1		12,332	33.5	
70–74	9992	29.6		11,021	30.0	
75–79	7651	22.7		8535	23.2	
80–84	3,758	11.1		3794	10.3	
≥ 85	1188	3.5		1117	3.0	

*SD* standard deviation, *KCL* the Kihon Checklist

**Fig. 2:**
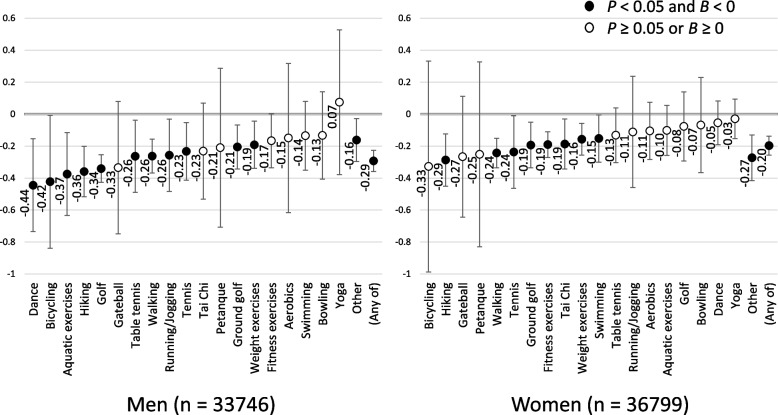
Unstandardized coefficients (*B*) of change in the Kihon Checklist total score according to each type of sport and exercise group participation after adjusting for age group, self-rated health, marital status, living alone, occupational status, years of education, alcohol consumption status, smoking status, equivalent income, and disease status using an inverse probability weighting method. Error bars represent upper and lower 95% confidence intervals

Figure 2 illustrates the unstandardized coefficient (*B*) and 95% CI values for the change in KCL scores for participation in each type of sports and exercise group compared to non-participation calculated by the IPW method. The sports/exercises that significantly prevented an increase in the KCL score for both sexes compared to non-participants were hiking (men: *B*, − 0.36; women: *B*, − 0.29), walking (men: *B*, − 0.26; women: *B*, − 0.24), tennis (men: *B*, − 0.23; women: *B*, − 0.24), ground golf (men: *B*, − 0.21; women: *B*, − 0.19), and weight exercises (men: *B*, − 0.19; women: *B*, − 0.16). For men, the sports/exercise types that significantly suppressed an increase in the KCL total score were dancing (*B*, − 0.44), bicycling (*B*, − 0.42), aquatic exercises (*B*, − 0.37), golf (*B*, − 0.34), table tennis (*B*, − 0.26), and running/jogging (*B*, − 0.26). For women, fitness exercises (*B*, − 0.19), Tai Chi (*B*, − 0.19), and swimming (*B*, − 0.15) were the activities that significantly prevented the worsening of total KCL scores.

Supplemental Table 2 shows the results for each interaction between the sports/exercise type and sex. They are listed in order of larger effect size for men than women. In particular, the effect size was larger for men than for women who engage in dance (*B*, 0.39), aquatic exercises (*B*, 0.27), and golf (*B*, 0.26).

## Discussion

To the best of our knowledge, this is the first longitudinal study to determine which specific types of sports and exercise groups influence frailty scores among community-dwelling older adults in Japan. While point estimates toward the suppression of increments in frailty scores were identified in almost all sports/exercise types, we observed that the relationship and effect size varied widely depending on the type of activity. Eleven sports/exercise types (led by dance) among men and eight sports/exercise types (led by hiking) among women were found to significantly reduce the worsening of frailty score compared to non-participants in each type of sport and exercise group. Considering that the frailty score worsened by an average of 0.6 points over the 3-year period (i.e., 0.2 points per year), participation in these types of groups had the equivalent effect of delaying frailty score worsening by approximately 1–2 years.

Although some of the sports/exercise types with significant associations were consistent among both sexes, others were not. Five sports/exercise activities (hiking, walking, tennis, ground golf, and weight exercises) were found to be effective in preventing frailty score increments regardless of the participant’s sex. While there was no consistency in the characteristics of these sports/exercises (such as the intensity of physical activity and competitiveness), all these activities were primarily performed outdoors, except for weight exercises. A previous systematic review indicated that exercising in a natural environment was associated with higher feelings of revitalization and positive engagement, a decrease in tension, confusion, anger, and depression, and increased energy compared to exercising indoors [22]. Thus, outdoor activities per se are likely to have a positive effect on the participant’s psychological well-being. Additionally, participants tended to report better enjoyment and satisfaction and declared a greater intent to repeat the activity. These characteristics of outdoor activities may particularly contribute to frailty prevention in older adults.

Weight exercises presumably contribute more directly to the maintenance of musculoskeletal function and have a positive impact on physical well-being, including the ability to perform activities of daily living. A well-designed resistance training program can counteract age-related changes in contractile function and the morphology of the aging human skeletal muscles, thus enhancing the individual’s muscle strength, power, and neuromuscular function [23]. Therefore, much evidence has proved that resistance training improves mobility, physical function, and performance in activities of daily living and contributes to the maintenance of independence in older adults [23].

The characteristics of the sport and exercise types that seemed to be effective in preventing frailty score increments, particularly in men, were relatively high-intensity aerobic and dynamic activities (such as dancing and cycling) and competitive games (such as golf and table tennis). Conversely, for the women, relatively moderate activities (such as fitness exercises and Tai Chi, the types of activities that increase self-awareness of their own bodies) were found to be effective. Previous longitudinal studies examining the association between 16 types of sports/exercise activities (regardless of group participation) and a decline in instrumental activities of daily living [24] and cognitive function [25] in older women confirmed that only calisthenics (synonymous with fitness exercises in the current study) effectively prevented functional decline for both outcomes. Some of these findings were also observed in our study. Although there are only a few studies examining the association between specific types of sports/exercises and health outcomes in older men, a previous study confirmed that older men’s participation in a golf group might have positive effects on various socio-psychological health statuses, including depressive symptoms, self-rated health, subjective well-being, and frequency of laughter [5]; none of these outcomes were significant in older women. A narrative review of sex differences in frailty status and the effect of interventions on frailty highlighted the potential to benefit from more sex-specific strategies alongside reporting the absence of strong supporting evidence [26]. We believe that the findings of the present study shall contribute to the existing literature. Further investigation is needed to identify the possible reasons for the observed sex differences in effectiveness.

Even though the estimated effect sizes were relatively large, there were some types in which no statistically significant effect was confirmed. This difference in statistical and clinical significance may be attributed to the fact that the number of participants in these activity types was relatively small and the 95% CI was expanded. If only participants of each of the different types were additionally sampled, they might have attained statistical significance. For example, bicycling (*n* = 42), gateball (*n* = 169), and petanque (*n* = 86) among women could have been significant if they had as many participants as those in tennis (*n* = 362), which was barely significant with a similar effect size. However, our results were based on a sufficiently large randomly sampled population, and we believe that they are of public health significance.

The strength of the current study is its large, nationwide, population-based longitudinal data enabling sex-stratified analyses for elucidating the frailty prevention benefits for 20 types of sports/exercise group participations among older adults. Nevertheless, the study also has the following limitations: First, we could not consider the quantity (e.g., frequency, duration, and intensity) and quality (e.g., social interaction, game properties, and competitiveness) of each sport/exercise group participation because of the restriction on the questionnaire. In addition, standard physical activity scales that have been comprehensively tested for reliability and validity were not used. Therefore, future investigations must consider these factors to infer which aspects of each type of sports and exercise contributed to the longitudinal association confirmed in this study and to discuss the underlying mechanism. Second, selection bias may have been introduced because the present study used data from participants who responded to the baseline (2016) and the follow-up (2019) surveys. This weakened the generalizability of the results and suggest that our sample underrepresented participants who were vulnerable to frailty, leading to an underestimation of the effect of participation in group sports and exercises. Lastly, we did not account for changes in the rates of participation in group sports and exercises and confounders during the follow-up period. Accordingly, we cannot adequately support the effects of continuing or interrupting participation in each type of group sport and exercise.

## Conclusions

Older adults who participated in hiking, walking, tennis, ground golf, or weight exercises in groups were less likely to experience worsening of their frailty scores compared to those who did not participate. We also found other types of sports/exercises that are expected to have sex-specific effects. Based on these findings, we recommend increasing group participation in the community to encourage these types of sports and exercises. We believe the results of this study offer substantive evidence to encourage older adults to participate in group activities for the prevention of multidimensional frailty. Our findings will also help public health stakeholders to decide which type of group sports and exercises to promote in a community.

### Supplementary Information

**Additional file 1: Supplemental Fig. 1.** Association between age and the total Kihon Checklist score in 2016. Error bars represent upper and lower 95% confidence intervals.

**Additional file 2: Supplemental Table 1.** Characteristics of the participants by sex (covariates).

**Additional file 3: Supplemental Table 2.** Results for the interaction between each type of sports/exercise and sex (*n* = 70,545).

## Data Availability

The data underlying this study are from the JAGES and contain sensitive information. Data for research purposes is available upon request. Requests for the JAGES data can be made to dataadmin.ml@jages.net.

## Abbreviations

IPW: Inverse probability weighting
JAGES: Japan Gerontological Evaluation Study
KCL: Kihon Checklist
