# Supplementary material for: Do changes in the frailty score differ by the type of group sports and exercises participated in? A 3-year longitudinal study
Source: Eur Rev Aging Phys Act. 2024 Mar 20;21:8. doi: 10.1186/s11556-024-00342-x (PMC10953207; doi:10.1186/s11556-024-00342-x)
Supplement: Supplementary file 1 — Additional file 1: Supplemental Fig. 1. Association between age and the total Kihon Checklist score in 2016. Error bars represent upper and lower 95% confidence intervals. [file 11556_2024_342_MOESM1_ESM.pdf]

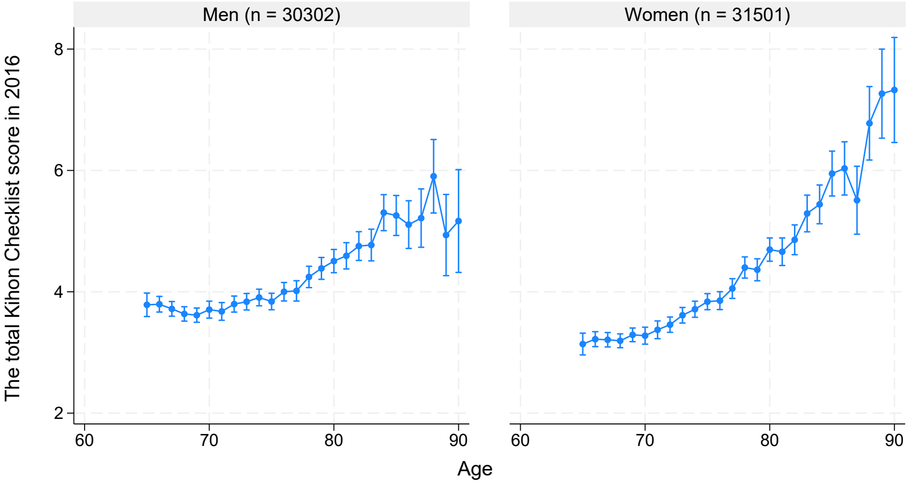

Supplemental Figure 1. Association between age and the total Kihon Checklist score in 2016.
Error bars represent upper and lower 95% confidence intervals.
